# Impact of Digital Transformation on Enterprise Carbon Intensity: The Moderating Role of Digital Information Resources

**DOI:** 10.3390/ijerph20032178

**Published:** 2023-01-25

**Authors:** Guoge Yang, Fengyi Wang, Feng Deng, Xianhong Xiang

**Affiliations:** 1 Xinjiang Innovation Management Research Center, Xinjiang University, Urumqi 830046, China; 2 School of Economics and Management, Xinjiang University, Urumqi 830046, China

**Keywords:** digital transformation, carbon emission intensity, information asymmetry, green innovation, digital information resources, financing constraints

## Abstract

In the context of China’s “digital power” strategy, the realization of a green and low-carbon shift in manufacturing has become a necessary condition to promote the economy, and the digital factor has increasingly become a new driving force. The text mining and IPCC methods were used to measure manufacturing enterprise digitalization and the level of enterprise carbon emission intensity from 2011 to 2021, respectively. This study then explored the impact of digitalization on manufacturing enterprise carbon emission intensity based on the least squares method model and instrumental variable method model. This research comes to three conclusions. (1) Digitalization can significantly reduce the enterprise carbon emission intensity of China’s manufacturing industry, and the influence shows a “marginal increase.” (2) Notably, a mechanism analysis indicates the intermediary effect sizes of four crucial intermediaries: green technology innovation > financing constraint > information asymmetry > energy use efficiency. Interestingly, digital information resources positively moderate the positive effect of digitalization on carbon emission intensity through three paths: financing constraints, green technology innovation, and information asymmetry. (3) The influence shows evident signs of heterogeneity—as environmental regulation, financial development, executive education, and R&D quality advance, the inhibiting effect of digitalization on enterprise carbon emission intensity becomes more pronounced. Finally, corresponding policy suggestions are proposed.

## 1. Introduction

Global carbon emissions continue to increase, resulting in climate warming, melting glaciers, and other environmental problems that have gradually threatened human health, equity, and sustainable development [1,2,3]. According to a survey by BP, China has been the world’s largest carbon emitter since 2005, with carbon emissions from the manufacturing sector increasing from 4.254 billion tons in 2005 to 8.386 billion tons in 2019, maintaining a growth rate of more than 10 percent. The digital economy has demonstrated strong resilience and great potential under the interwoven influence of the global pandemic and severe environmental concerns across the world [4]. The digital economy has provided unprecedented opportunities for China’s manufacturing transformation and low-carbon development [3,5]. In 1996, Tapscott first put forward the concept of the “digital economy,” believing that digital transformation would become the development trend of the future economy [3]. Subsequently, some scholars have stated that the digital economy mainly relies on information and communication technology to realize the economic trend of e-commerce transformation [6]. Some scholars have further argued that digitization not only realizes electronic commerce but also promotes business transformation through digital technology retail, drives the emergence of new markets and new forms of business, achieves economic growth, and increases enterprise labor productivity [7]. Therefore, enterprise digitalization not only contributes to the improvement of labor productivity but also improves traditional extensive modes of production and the allocation efficiency of innovative resources by enabling the production and R&D processes of enterprises through digital technology, thus enhancing green production capacity and innovation [8]. As the world’s second-largest digital economy, determining how China can use digital technologies to upgrade traditional R&D, production and management models, and promote the low-carbon transformation of its manufacturing industry is an important topic of research significance. As the main battlefield of China’s digital transformation, accelerating the digital transformation of the manufacturing industry has become a vital means to achieve low-carbon high-quality development in the manufacturing industry.

As the main issue of China’s digital transformation, determining how to accelerate the digitization of the manufacturing industry to reduce carbon emissions has concerned many scholars, but there is still much room for the analysis of associated realization mechanisms. There are still relatively few works that directly discuss digital transformation and carbon emission intensity, and most do not adequately discuss paths and channels of carbon reduction [9,10,11]. Based on information theory, this paper comprehensively analyzes the impact of digital transformation on carbon emission intensity from multiple paths, including information asymmetry and financing constraints. In addition, existing studies have discussed the influencing factors of reducing carbon emissions, but most focus on the effects of innovation [12,13], industrial structure adjustment [14], low-carbon pilot policies [15], financial development [16,17,18,19], and the introduction of FDI [20] and ICT [21] on the impact of carbon emissions. How exactly does digital transformation affect carbon intensity? What is the mechanism of its influence? Are any regional location and business environment conditions at play? To answer the above questions, text mining and the IPCC method were used to measure manufacturing enterprise digitalization and the level of enterprise carbon emission intensity from 2011 to 2021, respectively. This paper first explores the influence and mechanisms of digitalization effects on carbon intensity. We verify the reliability of our conclusions by using a series of methods, including the instrumental variable method and GGM method. In addition, based on information theory, we comprehensively discuss the mechanisms of digitalization, including carbon reduction from the four dimensions of information asymmetry, energy efficiency, and financing constraints, and we explore the regulatory role of digital information resources in each influence path. Finally, we further analyze the applicable conditions of digitalization: carbon reduction. The present research has important theoretical and practical implications for effectively releasing digital dividends and promoting the low-carbon transformation development.

The contribution of this paper is four-fold. First, regarding the rationality and scientificity of index measurement, most studies use the absolute value of enterprise carbon emissions for measurement, resulting in a lack of comparability of carbon emissions among enterprises [9,10,11]. This paper uses the carbon emission coefficient method to measure the relative carbon emission intensity of manufacturing enterprises as the explained variable to improve the scientific and rational measurement. This is because China advocates low-carbon transition development and attaches importance to the synchronization of carbon reduction and growth. Therefore, we have innovatively applied the IPCC method to the enterprise level to improve the rationality of measurement and scientific research. Second, regarding perspective innovation, text analysis methods are adopted to measure the digitalization of Chinese manufacturing enterprises, and on this basis, the paper discusses the impact mechanism of digitalization on the carbon emission intensity of manufacturing enterprises, enriching the literature on carbon emission factors. Third, regarding mechanism innovation, based on information asymmetry theory, this paper analyzes four paths, including information asymmetry, green innovation, energy efficiency, and financing constraints, and interestingly, it discusses the regulating effect of information accessibility, which deepens our understanding of the environmental effect path of the development of the digital economy. Finally, we explore heterogeneous innovation. Based on macro- and micro-level perspectives, we discuss the conditions and applicability of the impact of digital transformation on carbon emission intensity, providing more detailed evidence for the government to formulate emission reduction policies.

## 2. Literature Review

There are two main branches of literature that are closely related to this paper. One body of literature discusses the digital economy and green development, while the other discusses digitalization and enterprise low-carbon transformation development. For the digital economy and green development, some scholars take green TFP as a standard measurement of green development and find that the digital economy can improve green TFP by optimizing industrial structures [22] and strengthening factor resource allocation [23]. Other scholars interpret the connotations of green development from different perspectives. They explore the path mechanisms of the digital economy affecting environmental quality and green innovation, finding that the digital economy can promote regional green innovation by increasing investment in R&D and human resources [24]. Such an approach can also contribute to the environmental improvement of local and neighboring areas [25]. Other scholars have focused on the environmental consequences of raw material and power consumption in manufacturing and operations and from the disposal of digital devices and services [26]. Such scholars find that digital technology is a double-edged sword for energy consumption and carbon emissions. On the one hand, the application of traditional ICTs can directly drive energy demand, especially for fossil fuels, causing carbon emissions associated with energy use to rise simultaneously [27]. On the other hand, improving energy efficiency through the use of ICTs also has the potential to trigger a rebound effect, leading to higher carbon emissions [28]. In addition, a small number of scholars find that the digital economy on green development presents a nonlinear relationship [29,30].

Another strand of literature focuses on digitization and the development of the low-carbon transformation of enterprises. Some scholars believe that digitalization can promote the rational allocation of internal resources of enterprises and enhance green innovation through an information effect [31,32]. However, others argue that digitalization requires installing more energy-intensive computers, which could increase carbon emissions [33]. A few scholars also disagree with the idea that enterprise digitalization and carbon emissions only have a linear relationship and verify that there may also be an inverted U-shaped relationship between the two [34]. On this basis, some studies have discussed in depth the existence of a threshold value whereby only within this threshold can digital transformation be conducive to green development [35]. Therefore, not all enterprises are suited to digitalization, and the carbon reduction effect of digitalization should not be exaggerated; otherwise, it may not be conducive to low-carbon development [36]. In summary, scholars have not reached a consensus on the relationship between digital technology and low-carbon and green development.

There are some deficiencies in the existing literature. First, most research data focus on macro-level examples. The discussion of macro-level carbon emissions is also based on the total carbon emissions of all microenterprises, while it may ignore the emission characteristics of microenterprises and lack micro-level guidance. Second, although a few areas of literature analyze digital environmental effects from a microscopic perspective, most discuss the impact on enterprises’ green innovation and total factor productivity, and few directly measure the carbon intensity of Chinese manufacturing enterprises. Finally, the mechanism through which corporate digital transformation affects corporate low-carbon development is not yet sufficiently detailed. Based on this, this paper measures the digitalization index of Chinese manufacturing enterprises and analyzes the mechanisms of how digitalization transformation affects enterprises’ carbon intensity based on information theory and financing constraint theory. It is found that digitalization can not only accelerate the restructuring of the manufacturing industry but also improve the energy efficiency of enterprises. Moreover, digitalization can help enterprises achieve low-carbon transformation by easing the risk of information asymmetry between enterprises and external stakeholders and alleviating financing constraints.

## 3. Theoretical Analysis and Hypothesis

### 3.1. Digitalization and Information Asymmetry

According to information theory, market information asymmetry will lead to market risk and then reduce the efficiency of market operations, which is not conducive to the optimal allocation of resources [37,38]. On the one hand, digital technology can increase exchanges and communication between different subjects, strengthening the knowledge spillover effect of low-carbon technologies [39]. On the other hand, digital development strengthens the links between regional industries, alleviates the analysis of information asymmetry between regions, reduces the market segmentation behavior of local governments with the main motive of protecting the local market, and effectively alleviates the mismatch of information resources at the spatial and industrial levels [40,41,42]. In addition, digital technology can improve regional market integration and promote the optimal allocation of resources and factors between regions by reducing the barrier effects of geographic distance on price information transmission [43], which will help reduce transportation and market transaction costs, and alleviate the carbon overload caused by repeated construction [44,45]. Digital transformation can help enterprises realize the real-time acquisition and transmission of information over long distances using digital means, break information islands, and improve information accessibility between internal and external enterprises as well as between enterprises [46,47]. On the one hand, it is helpful for enterprises to obtain more green resources externally to support and improve their green development abilities, and it is also helpful to strengthen the exchange of experiences between enterprises involved in the same areas of trade and enhance cooperation in carbon emission research and development between enterprises [48,49,50]. On the other hand, digital development provides all enterprises with access to the Internet, creating the opportunity to obtain diversified green financial services at low costs, and providing resource support for enterprises’ carbon emission reduction [51,52]. Hence, we propose Hypothesis H1.

**H1.**  *Information asymmetry mediates the impact of digital transformation on enterprise carbon intensity*.

### 3.2. Digitalization and Energy Efficiency

China’s low energy efficiency is the result of extensive production methods. China’s extensive business model is an important cause of carbon overloading. The existing studies have found that enterprises’ digital transformation can reduce carbon emissions by improving energy efficiency [53,54,55]. On the one hand, digital technology is applied to optimize the production process and efficiency. The main reason for China’s traditional mode of high consumption and high emission is the backward production process and production mode. The application of digital technology can help to promote the transformation of the production mode of the traditional manufacturing industry, not only improving the labor productivity of enterprises but also improving the efficiency of energy and resource utilization, thus reducing the carbon emission intensity of enterprises [56,57,58]. Some scholars have found that industries with a high degree of digitalization have lower carbon emissions [59,60,61]. On the other hand, digital technology comes from ICT, which is also recognized as an effective means of managing a country’s energy needs and helping reduce energy use [27]. Therefore, enterprise digital transformation can help promote the transformation of enterprise energy consumption structure, improve the resource utilization rate, and reduce enterprise carbon emission intensity [62]. Based on this analysis, we propose Hypothesis H2.

**H2.**  *Enterprise energy efficiency mediates the impact of digital transformation on enterprise carbon intensity*.

### 3.3. Digitalization and Green Innovation

The core driving force of digitalization is information technology. The most significant impact of the information technology revolution on economic activities lies in its provision of labor productivity and promotion of specialization [63,64]. Therefore, China’s innovation activities are driven by the professional advantages of digital technology [65]. On the one hand, the high permeability and applicability of digital technology can promote industrial transformation and upgrading. On the other hand, cross-industry digital technology spillover enables regional low-carbon technology innovation and continuously reduces the carbon emissions of enterprises in the same region [66].

First, digital technology has profoundly changed people’s way of thinking. Through digital sustainability activities, we can stimulate an internal entrepreneurial spirit, enhance the green innovation ability of enterprises, and balance social development and ecological protection [67]. Second, the development of the Internet will force enterprises to pay attention to ecological protection while continuing to innovate. That is, digital technology can broaden the depth of green innovation through a reversal mechanism. Meanwhile, digitalization is the product of technological innovation, which comes with cumulative and iterative effects of technology. Such effects can constantly break through the boundaries of fintech through the application of carbon trading, carbon capture, carbon utilization, and carbon sequestration, promoting the continuous innovation of green technology [68]. Finally, digitalization accelerates the innovative development of financial products, which is conducive to the green R&D activities of enterprises [69]. This integration will help better bridge the gap in traditional finance, promote the diffusion of financial resources to technology-oriented enterprises, and enhance enterprises’ green technology innovation capabilities [70], helping reduce carbon emissions. Hence, we propose Hypothesis H3.

**H3.**  *Green innovation mediates the impact of digital transformation on enterprise carbon intensity*.

### 3.4. Digitization and Financing Constraints

Information asymmetry will prevent stakeholders from providing innovative investment or credit support to enterprises, thus easing corporate financing constraints [71]. Studies have shown that strong financing constraints will lead to a reduction of enterprise capital and labor input and distort enterprise resource allocation [72]. On the one hand, digital development can reduce the financing information costs of enterprises, enrich financing channels, and improve financing efficiency, helping ease financing constraints and improve low-carbon development [73,74,75,76]. FDI, on the other hand, tends to flow into financially stable countries [77]. Digitalization can effectively improve the efficiency of financial services, alleviate the systemic risks of traditional finance, reduce the uncertainties of investment, and promote the building of a high-quality, stable, and safe business environment, thus continuously attracting FDI inflows [78]. Therefore, the technology spillover effect brought by FDI is conducive to promoting the study and imitation of Chinese enterprises to reduce carbon emission intensity [20,79]. In summary, the digital economy can help ease enterprise financing information, improve enterprise resource abundance, and realize the sustainable development of the green finance industry [80], which is beneficial to reducing enterprise carbon emissions. Based on this analysis, we propose Hypothesis H4.

**H4.**  *Financing constraints mediate the impact of digital transformation on enterprise carbon intensity*.

### 3.5. The Moderating Effect of Digital Information Resources

The fundamental feature of the digital economy is connectivity. The digital economy uses information elements as a source of productivity, information technology as a driving force, and information networks as the carrier. Through the connections and interaction between data elements and traditional production factors, the digital economy will help fundamentally change the technology base and business models of traditional manufacturing industries, remove limitations of time and space to connect various industries and regions, and achieve an optimal allocation of resources by alleviating information asymmetry. In addition, China’s current fiscal decentralization system leads to regional industry segmentation, resulting in the segmentation of the market, reducing information efficiency, and making regional green and low-carbon development unfavorable [40,47,81]. The rich digital information resources based on the Internet realize the real-time transmission of information over long distances, greatly improve information accessibility among regions, industries, and enterprises, and alleviate market information asymmetry defects. Therefore, we believe that the richness of regional digital information resources directly affects the digital transformation of enterprises. Based on the above analysis, we propose Hypothesis H5.

**H5.**  *Digital information resources moderate the impact of digital transformation on enterprise carbon emission intensity*.

**Figure 1:**
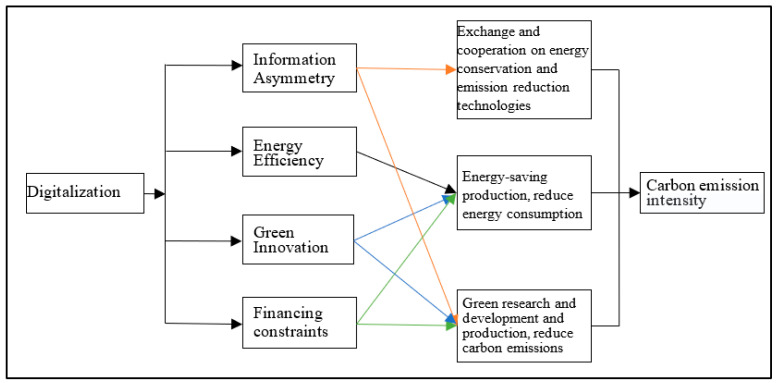
Mechanism diagram of the impact of digitalization on carbon emission intensity.

Based on the above analysis, the digital transformation of enterprises mainly reduces carbon emission intensity by reducing information costs, changing the production mode of enterprises, and improving the level of green research and development. To better demonstrate the mechanical framework, we use the conceptual model to summarize, as shown in Figure 1.

## 4. Model Settings, Variables, and Data Sources

### 4.1. Model Setting

This paper discusses the impact of digital transformation on enterprises’ carbon intensity. The least square regression model is constructed to discuss the influencing factors of carbon intensity. Referring to the practice of established scholars [5,8,82], the specific model is shown in (1):

(1) $$ECOEMISQ_{it}=\alpha_{0}+\alpha_{1}digital_{it}+\alpha_{c}X_{it}+i.company_{i}+i.year_{t}+\varepsilon_{it}$$

In Model (1), ECOEMISQ indicates the carbon intensity of enterprises, and digital is the digital transformation index of enterprises. The vector *X_it_* represents a set of control variables, company stands for individual fixed effect and Year represents time fixed effects, *ε_it_* said random disturbance.

### 4.2. Variable Measure and Description

#### 4.2.1. Enterprise Carbon Emission Intensity

Carbon emission intensity refers to the carbon dioxide emission brought by each unit of GDP, that is, carbon emission intensity = carbon emission/GDP. The index is used to measure the relationship between a country’s economy and its carbon emissions. A country has achieved a low-carbon development model if its carbon dioxide emissions per unit of gross national product are falling while its economy is growing. This paper mainly adopts the IPCC method. In terms of data availability, nine energy sources are mainly selected as measurement indicators in this paper. We first need to obtain the raw energy consumption data of China’s manufacturing industry through the China Energy Statistical Yearbook and then convert the energy consumption data into standard coal. The conversion coefficient and carbon emission coefficient of various energy standard coal are shown in Table 1.

The first step is to calculate the carbon emissions of the manufacturing industry. The formula is shown in (2).

(2) $$COEMIS_{t}=\sum_{i=1}^{12}Ci=\sum_{i=1}^{12}AiBiDi\times \frac{44}{12}$$

where *Ci* is the carbon emission of the I-th energy; *Ai* is the I energy consumption; *Bi* is the coefficient of converting the I-th energy into standard coal; *Di* is the carbon emission coefficient of the I-th energy; the constants *Ai* and *Bi* are from China Energy Statistical Yearbook; the constant *Di* is derived from the 2006 IPCC National Greenhouse Gas Guidelines, and 44/12 is the conversion factor of carbon emissions to CO_2_ emissions.

The second step is to calculate the enterprise’s carbon emissions.

(3) $$ECOEMIS_{i}{}_{t}=\frac{eincom}{indsincom}\times COEMIS_{i}{}_{t}$$

*COEMIS_it_* refers to the carbon dioxide emissions of enterprise *i* in *t* years, which is equal to the product of the ratio of business income of the enterprise in the current year to the main business income of the industry and the carbon emissions of the industry. *INCOME_it_* represents *t* year operating income of enterprise *i*, and *MINCOME* represents *t* year main business income of the manufacturing industry.

The third step is to calculate the carbon emission intensity of enterprises. *EGDP_it_* represents the total output value at the end of the year. The calculation is shown in Formula (4).

(4) $$ECOEMISQ_{i}{}_{t}=\frac{ECOEMIS_{i}{}_{t}}{EGDP_{it}}$$

#### 4.2.2. Digitalization Index

Some scholars have statistically analyzed the word frequency of digital economy keywords in the annual reports of listed companies through crawler technology and used it to describe the degree of digital transformation of enterprises that have been recognized by academic circles [8]. Annual reports between different companies are summarizing and forward-looking in nature, so the digital index between listed companies is comparable. Therefore, this paper uses the statistical method of word frequency of digital economy keywords to construct the digital index of manufacturing enterprises. Specific steps: first, make clear the keyword source of digital transformation. This paper refers to the relevant policy documents, research reports, and the enterprise digital empowerment action plan issued by the General Office of the Ministry of Industry and Information Technology. Secondly, the crawler technology is used to download the annual reports of enterprises, extract the word frequency related to digital transformation and build a digital dictionary for this paper [8,9]. On this basis, Python Chinese word Segmentation Library (jieba) is used for word segmentation. Thirdly, the digital keywords of each listed company are frequently added to get the digital economy index. Finally, the ratio of the total digital word frequency to the total number of words in the annual report is used as the enterprise digitalization index (digital). As shown in formula (5). And use the WINSOR2 command of STATA software to reduce the tail of the variable at the 1% level.

(5) $$\mathrm{digital}\frac{\mathrm{Total}\;\mathrm{number}\;\mathrm{of}\;\mathrm{digital}\;\mathrm{keyword}\;\mathrm{frequencies}}{\mathrm{Total}\;\mathrm{number}\;\mathrm{of}\;\mathrm{words}\;\mathrm{in}\;\mathrm{the}\;\mathrm{annual}\;\mathrm{report}}$$

#### 4.2.3. Mechanism Variables

(1) Asymmetric information (lnasymme): We use the ratio of absolute manipulative accruals to total assets of listed companies to represent the information asymmetry.

(2) Energy utilization rate (energy): In this paper, the energy consumption rate is used as the reverse proxy index of energy utilization. Energy consumption rate: We use the ratio of coal consumption and total fuel consumption to the gross product of the enterprise as the proxy index of the energy consumption rate.

(3) Green innovation (patent): We used green patent invention combing as a proxy variable

(4) Financing constraints (ln*SA*): The representative measurement methods include KZ index, WW index, and SA index, but the three indexes all have defects to some extent. The existing research practices in this paper use the improved SA index [83,84]. Specific calculation:

(6) $$SA=-0.737\times size+0.043\times {\left(size\right)}^{2}-0.040\times age$$

In Formula (6), the *size* represents the total assets of the enterprise, and *age* represents the years of operation of the enterprise. *SA* is always negative, so we’re dealing with absolute values to prevent outliers from interfering with our natural logarithm processing of the mechanism variables

#### 4.2.4. Control Variables

Control variables of this paper: (1) enterprise age (range). We use the establishment years of enterprises as the proxy variable. (2) Enterprise scale (in size): represented by the number of employees. (3) Profitability (income): represented by the current operating income of the enterprise. (4) Enterprise cash flow (in cash): we use corporate cash representation. In addition, we also control some of the industry influences. (5) Industrial competition (HHI): our Herfindahl index represents the level of competition in the industry. (6) Productivity of industry (TFP): we adopt the industry total factor productivity measured by DEA. To prevent heteroscedasticity interference, natural logarithm processing was carried out for control variables at the enterprise level.

### 4.3. Data Description and Descriptive Statistical Analysis

The A-share listed companies in Shanghai and Shenzhen during 2011–2021 are selected from the CSMAR database, and 13–43 categories of C categories of manufacturing are selected according to industry codes and according to the standard of Industry Classification of National Economy (GB/T4754-2012). Financial enterprises and ST samples were deleted considering missing values and other reasons. Non-equilibrium panel data from 2030 companies in 11 years were obtained, with a total of 12,426 sample observations. Table 2 reports descriptive statistical results. The results show that the mean value of ECOEMISQ in the manufacturing industry is −0.0210, the maximum value is 2.8625, and the minimum value is −1.9150. The standard deviation is 0.2182. The mean value of enterprise digital is 0.4054, and the standard deviation is 0.2473. The maximum value is 0.8673 and the minimum value is 0.0305. Whether digital transformation can reduce carbon emission intensity needs further empirical analysis.

## 5. Empirical Analysis

### 5.1. Benchmark Regression

The estimation results in Table 3 demonstrate the effect of the level of digitalization of enterprises on carbon emission intensity. Column (1) shows that digitalization has a significant inhibiting effect on the carbon intensity of enterprises without considering other control variables. The estimation results in columns (2) to (7) progressively add control variables to the estimation results. Column (7) reports the estimation results that control for individual and time and include all control variables, showing that the effect of corporate digitalization on carbon intensity is significantly negative at the 1% statistical level. It indicates that digital transformation still contributes to reducing the carbon intensity of enterprises. This is consistent with the expected hypothesis [10,85,86,87,88]. Compared with previous studies, we conclude that digital transformation has a greater carbon reduction effect [82,89]. There may be two reasons for this. First, most of the previous studies used data from the provincial, city, and listed companies, which was not very targeted [53,88,90]. Second, most of them used the absolute value of carbon emissions or the composite index as the proxy variable, which may cause the comparability and economic significance of the estimated results to be unclear [89,91,92]. According to the reality of China, low-carbon transformation development requires carbon reduction and growth at the same time, so environmental regulation policies need to take into account both corporate economic effects and environmental effects. For city or provincial data, estimation results provide directional and strategic guidance, but for enterprise data, we believe that accurate estimation results are more conducive to policy making. Therefore, relative carbon emission intensity is used in this paper, which improves the scientific index measurement and comparability of estimation conclusions.

The estimation results of control variables show that the effect of enterprise age is negative, which may help to control enterprise carbon emissions by increasing technological innovation and energy use of enterprises. The effect of enterprise size is positive and statistically insignificant. The effect of enterprise profitability is significantly positive at the 1% statistical level, possibly because China’s business model expansion is generally sloppy, and gaining more profits by expanding production scale may exacerbate carbon emissions. Corporate cash flow helps to curb carbon emission intensity. At the industry level, the more competitive the industry is, the more favorable it is for enterprises to reduce carbon emissions. This may be because a competitive market will force enterprises to research and develop innovations. There is a negative relationship between industry productivity levels and enterprises’ carbon emission intensity, indicating that higher industry productivity helps enterprises reduce carbon emissions. This is because technology spillover effects within and between industries help enterprises learn and improve productivity.

### 5.2. Robustness Test

#### 5.2.1. Outlier Handling

Measurement errors may exist in the data collection process. Considering the interference of outliers may lead to bias in the estimation results. We perform outlier treatment to remove outliers. In this paper, we do the tailing treatment for the variables at 1% and 99% levels. The test results are shown in column (1) of Table 4. The estimation results are still robustness. In terms of coefficients, the suppression effect is greater, which may be due to outlier interference, but the overall significance and sign are consistent with the baseline regression.

#### 5.2.2. Replacement Estimation Method

Considering that the estimation sample in this paper is a restricted subset of the overall manufacturing enterprises, it may cause biased estimation, so we choose the restricted dependent variable estimation method for re-estimation. As is shown in column (2) of Table 4, there is a significant negative relationship between enterprise digitalization and carbon emission intensity, which is consistent with the results estimated using the least squares method. It shows that the inhibitory effect of enterprise digitalization on carbon emission intensity is not disturbed by severe sample bias.

#### 5.2.3. Substitution of Explanatory Variables

It is a scientific and feasible method to measure the degree of enterprise digitalization through digital word frequency. According to the research of existing scholars, digital assets are also intangible assets. Therefore, we used the proportion of digital intangible assets as a proxy variable to test. Specifically, this included “software”, “network”, “client management system”, “intelligent platform” and other digital technology-related keyword statistics and sums. By using digital technology, the total value of intangible assets and intangible assets at the end of the year represent the digitalization degree of an enterprise. The calculation formula is shown in (7).

(7) $$\mathrm{digital}=\frac{\mathrm{Total}\;\mathrm{intangible}\;\mathrm{assets}\;\mathrm{of}\;\mathrm{digital}\;\mathrm{technology}}{\mathrm{Total}\;\mathrm{intangible}\;\mathrm{assets}\;\mathrm{of}\;\mathrm{the}\;\mathrm{enterprise}\;\mathrm{at}\;\mathrm{the}\;\mathrm{end}\;\mathrm{of}\;\mathrm{the}\;\mathrm{year}}$$

The regression results in column (3) of Table 4 indicates that accelerating the digital transformation of enterprises can help reduce the carbon emission intensity of enterprises. The robustness of this paper is verified.

#### 5.2.4. Replacing the Explained Variable

Considering that the relative carbon emission intensity of the enterprise covered up the carbon emission information of the data, we used absolute carbon emission intensity as a proxy variable to conduct the robustness test. In order to eliminate heteroscedasticity, the absolute carbon emission intensity is treated logarithmically. The estimated results are shown in column (4) of Table 4. The results show that the impact of digital transformation on the absolute carbon intensity of enterprises is significantly negative at the statistical level of 5%, indicating that the digital transformation of enterprises can help reduce the absolute carbon intensity of enterprises. This proves the robustness of the conclusion to a certain extent.

#### 5.2.5. Handling Omitted Variables

We include the level of enterprise R&D and financial leverage. We use the ratio of R&D investment expenses to operating revenue as a proxy variable for the level of technological innovation of the enterprise (rd). Corporate financial leverage is used as a proxy variable for the financial position (lev). The results of adding omitted variables to the model are shown in column (5) of Table 4. The results indicate that the findings of this paper do not have strict omitted variables.

### 5.3. Endogeneity Test

To address the endogeneity issue, this paper draws on the approach of established scholars [43,91]. We first construct the instrumental variables for enterprise digitalization using the city 1984 digital circuit and the city Internet user cross multiplication. Again, city data is used to match with enterprise data. Because the city’s historical digital infrastructure construction has had a significant impact on the development of the city’s digital economy, and thus on the digital transformation of the enterprise, the instrumental variables therefore satisfy the relevance requirement. The level of enterprise digitalization does not affect the historical infrastructure development of cities, and thus satisfies the homogeneity requirement.

Column (1) of Table 5 shows that the instrumental variables are significantly correlated with digitalization. and passed the first-stage F-value test. Column (2) shows that after addressing endogeneity, digital transformation can still significantly suppress the carbon emission intensity of enterprises. In addition, the estimated model and omitted variables are considered to cause the endogeneity problem. Columns (3) and (4) report the results of systematic GMM and differential GMM estimation. After passing the AR2 and Hansen tests, it shows that the instrumental variables are chosen reasonably. The conclusions of this paper remain robust after the endogeneity test.

## 6. Heterogeneity Analysis

### 6.1. Environmental Regulation

Environmental regulation policies vary from region to region. Currently, there are market-based environmental regulations such as carbon trading. There are also imperative environmental regulations for carbon emission reduction. Regardless of the type of environmental regulation, it will affect the carbon emission intensity of regional enterprises. We divide cities into environmental regulation cities and non-environmental regulation cities according to environmental regulation. Then we match with enterprises according to their registered cities. The results of subgroup estimation are shown in Table 6. Columns (1) and (2) show the regression results of the impact of enterprise digitalization on carbon emission intensity moderated by environmental regulation. The results show that the suppression effect of corporate digital transformation on carbon intensity is more significant in environmentally regulated cities. Environmental regulation policies may help to push back the digital transformation of enterprises and strengthen the carbon reduction effect of digital transformation. In areas without environmental regulation, the digitalization of municipal enterprises still has a positive inhibitory effect on carbon emission intensity, but it is relatively weaker. Therefore, enterprises themselves shoulder the main responsibility of reducing pollution and carbon, but appropriate environmental regulation policies formulated by external government departments can help improve the environmental effects of digital transformation, -ultimately realize low-carbon transformation development. Additionally, by using the between-group estimation test, the *p*-value is found to be equal to 0.0001, indicating that there is a significant difference between the coefficients of the groups. It indicates that the government pays attention to carbon emissions and puts pressure on the carbon emissions of regional enterprises, which helps to ultimately promote regional low-carbon development. Economic-type environmental regulation displays the characteristics of a market economy by determining ownership, while the main core idea of command-type environmental regulation is the transformation of service-oriented government.

### 6.2. Level of Financial Development

Resource-based theory suggests that enterprises’ access to abundant resources is the basis of enterprise innovation. The abundance of corporate financing resources may directly affect the energy-saving and emission-reduction behaviors of enterprises. Therefore, it is of great significance to test the influence of the abundance of financial resources on enterprises’ digital transformation. Firstly, we use the ratio of city deposit and loan amount to city GDP to characterize the level of financial development. Secondly, the city data is matched with the enterprise data by using the information from the enterprise registration location and the mean values are used to group. Table 6 shows that in the high-level group, the effect of enterprise digitalization on carbon emission intensity is significantly positive. It indicates that the higher level of financial development has a greater inhibitory effect of corporate digital transformation on carbon emission intensity. Regional resource endowment or regional location will affect the business environment of the region, especially the level of financial development for an enterprise development bailout. The government should actively optimize the business environment, accelerate financial development, attract more high-quality investment companies or financial companies to settle in, and diversify financing channels.

### 6.3. Quantile Test

The least squares estimation method assumes that digitalization can only affect the mean information of the conditional distribution of carbon intensity, which cannot fully explain the suppressive effect of digitalization on carbon intensity. To explore in more detail the variability of the effect of digitalization on carbon intensity at different categorization levels, the article performs quantile regressions on carbon emission intensity levels at the 25%, 50%, 75%, and 99% quantile levels. Column (1) in Table 7 shows that the effect of digitalization on the carbon intensity of enterprises is negative but statistically insignificant at the 25% quantile. As the quantile level increases, the negative effect of digitalization is greater. Quantile regression can make up for the shortcomings of the least squares method and more accurately evaluate the impact of digitalization on the carbon emission intensity of enterprises. By quantile regression, we can find the marginal increasing effect. In other words, when the relative carbon emission intensity of enterprises increases, the inhibitory effect of digital transformation on enterprise carbon emission becomes more obvious, in order to provide more reliable estimation conclusions for policy formulation and implementation.

### 6.4. Educational Background of Executives

Entrepreneurs are the driving force behind corporate innovation, and the decision-making ability of entrepreneurs is directly related to the sustainable development of enterprises. Therefore, the educational background of entrepreneurs may affect the speed and quality of digital transformation of different enterprises. Because education at the undergraduate level and below is general education, it does not require much thinking about students’ R&D abilities. Education at the graduate level and above mainly cultivates students’ research thinking. Therefore, we speculate that different educational backgrounds will have different impacts on the digital development and low-carbon development of enterprises. We test the grouping based on the education of corporate chairmen and general managers. We define enterprises whose chairman and general manager’s degrees are both master’s degrees or higher as the high education group, and enterprises whose chairman and general manager’s degrees have at least one educational background of bachelor’s degree or lower as the low education group. The results of the subgroup estimation are shown in Table 8. Columns (1) and (2) show that there is variability in the effect of digitalization on carbon emission intensity in terms of the educational background of business leaders. Column (1) indicates that with higher education, companies may have a greater awareness of R&D, which helps to strengthen the inhibitory effect of digital transformation on carbon emission intensity. Therefore, in the digital transformation of enterprises, one must pay attention to the introduction of talents with high educational backgrounds. In addition, local governments have attached importance to education, increasing the admission rate of graduate students, increasing support for the training of digital professionals, and providing intellectual support for the development of enterprises.

### 6.5. R&D Quality

There are significant differences in knowledge resource endowments within different enterprises, which may lead to variability in enterprises’ accumulation of low-carbon R&D and green innovation, resulting in differences in low-carbon development. We adopt the number of patent applications as a proxy variable for enterprise knowledge resources. Patent invention applications include invention patents, utility, and appearance patents. The quality of invention patents is higher than that of utility and appearance patents. Therefore, we divided the sample into a high R&D quality group and a low R&D quality group according to the level of patent quality. The estimation results of the subgroups are shown in columns (3) and (4) in Table 8. Column (3) indicates that in the low R&D group, digital transformation is instead detrimental to the carbon emission reduction of enterprises. It may be because the enterprises’ low-quality innovation did not implement carbon emission reduction measures to cope with the policy pressure. The estimation result of column (4) indicates that encouraging enterprises to improve the quality of patent inventions can help strengthen the inhibitory effect of digital transformation on carbon emission intensity and promote low-carbon development. To establish a more reasonable and strict patent application system and mechanism to provide a basis for improving patent quality, the government has vigorously encouraged enterprises to innovate and rewarded high-level R&D enterprises, forming a sound incentive mechanism for R&D subsidies.

## 7. Mechanism Test

### 7.1. Mediation Model Setting

To further explore how digitalization affects the carbon emission intensity of enterprises, we conducted a mechanism test. According to the previous analysis, digitalization development will suppress the carbon emission intensity of enterprises by alleviating information asymmetry and financing constraints, improving energy use efficiency and the green technology innovation level. We tested this by constructing a mediating mechanism model [93]. The mediating effect model is shown in Equations (8)–(10).

(8) $$Mechanism_{it}=\beta_{0}+\beta_{1}digital_{it}+\beta_{c}X_{it}+i.company_{i}+i.year_{t}+\varepsilon_{it}$$

(9) $$ECOEMISQ_{it}=\gamma_{0}+\gamma_{1}Mechanism_{it}+\gamma_{c}X_{it}+i.company_{i}+i.year_{t}+\varepsilon_{it}$$

(10) $$\begin{array}{c} ECOEMISQ_{it}=\alpha_{0}+\alpha_{1}digital_{it}+\alpha_{2}Mechanism_{it}+\alpha_{c}X_{it}\\ +i.company_{i}+i.year_{t}+\varepsilon_{it} \end{array}$$

Model (8) Mechanism denotes mechanism variables, including information asymmetry, energy utilization, green technology innovation, and financing constraints. ECOEMISQ in model (9) denotes the carbon emission intensity of enterprises. Model (10) is based on model (1) by adding mechanism variables, and the parameters are consistent with the interpretation of model (1).

### 7.2. Intermediate Effect Test

#### 7.2.1. Information Asymmetry

The digital information dissemination method based on the Internet realizes the instantaneous dissemination of information over long distances, which significantly improves the accessibility of information between regions, industries, and enterprises, and alleviates the risk of incomplete information for enterprises. Therefore, improving the digital level of enterprise enhancement helps to alleviate the risk of information asymmetry and strengthen the exchange and cooperation of energy-saving and emission-reduction technologies among enterprises. Thus, information asymmetry may play a mediating role. We drew on established studies to substantiate this [94]. The ratio of the absolute value of manipulative accruals to total assets of listed companies is used to characterize information asymmetry. The estimated result shows that digitalization has a significant inhibitory effect on information asymmetry in column (1). Column (2) shows that the effect of information asymmetry on carbon emission intensity is significantly positive, indicating a significant positive relationship between them. Column (3) is also added to the model for regression, and it is found that the digital transformation of enterprises is significantly negative. It indicates that the digitalization of enterprises can reduce the carbon intensity of enterprises by reducing information asymmetry. Information asymmetry plays the role of a mediating mechanism, and Hypothesis H1 is verified.

#### 7.2.2. Energy Utilization

We used the energy consumption rate as a reverse proxy for energy utilization. For the energy consumption rate, we used the ratio of total enterprise coal consumption and fuel consumption to enterprise GDP as a proxy for the energy consumption rate (energy). A larger value indicates a lower energy utilization rate, and conversely, a smaller value indicates a higher energy utilization rate. The estimated results in column (4) of Table 9 show that the digital transformation of enterprises can significantly suppress the energy consumption rate of enterprises. Column (5) shows that there is a significant positive relationship between energy consumption rate and carbon emission intensity. Column (6) estimates show that digital transformation remains significantly negative and that the energy consumption rate is significantly and positively correlated with the carbon emission intensity of enterprises. In summary, it can be shown that energy utilization plays a mediating role in the digital transformation of enterprises to reduce the carbon emission intensity of enterprises, and Hypothesis H2 is verified.

#### 7.2.3. Green Technology Innovation

We used the number of green patents as a proxy variable (lngreenpatent) for green technological innovation. The results of the mechanism test are shown in Table 10. Columns (1) to (3) report the estimation results of the mediating effect of green technology innovation. The estimation results in column (1) show that there is a significant contribution of enterprise digitalization to enterprise green patents. Column (2) shows that the effect of green technology innovation on corporate carbon emission intensity is significantly negative. The estimation results in column (3) indicate that digital transformation can reduce corporate carbon emission intensity by promoting corporate green technology innovation. Therefore, green technology innovation is an important path to reduce carbon, and the existing literature has drawn similar conclusions [95]. Hypothesis H3 is verified.

#### 7.2.4. Financing Constraints

Digital technology development has also brought a new paradigm of ICT in the financial industry to promote financial innovation—digital finance. On the one hand, digital finance development broadens financing channels and lowers financing thresholds to ease corporate financing constraints. On the other hand, digital economy development helps to alleviate the risk of information asymmetry of enterprises, mitigate the risk of asymmetry between enterprises and external stakeholders, and improve financing efficiency. When the enterprise financing constraint is reduced, it is conducive for enterprises to spend more resources on emission reduction technology and industrial structure upgrading [84]. The estimation results are shown in Table 10. Column (4) shows that the effect of enterprise digitalization on financing constraints is significantly negative at the 1% statistical level. It indicates that digitalization is negatively related to financing constraints and increasing the digitalization level of enterprises helps to reduce their financing constraints. Column (5) shows that the financing constraint index has a significant positive influence on the carbon emission intensity of enterprises. The estimation results in column (6) show that financing constraints play an important role in the operation of intermediary mechanisms. Hypothesis H4 is verified.

### 7.3. Further Analysis

#### 7.3.1. Moderating Model

According to the theoretical analysis, regional digital information resources serve as the foundation of enterprise digital transformation and provide digital resource support for digital transformation. To test, whether digital information resource regulation positively moderates the effect of enterprise digital transformation on carbon emission intensity, we constructed the moderation effect model, as shown in Equation (11).

(11) $$\begin{array}{c} ECOEMISQ_{it}=\alpha_{0}+\alpha_{1}digital_{it}+\alpha_{2}digital_{it}\times access_{it}+\alpha_{3}access_{it}+\alpha_{c}X_{it}\\ +company_{i}+year_{t}+\varepsilon_{it} \end{array}$$

Model (11) access represents the moderating variable of digital information resources. Model (11) is based on model (1) with the addition of digital information resources and digital interaction terms. We focused on the interaction term coefficient α3 sign positive or negative and significance. Significantly positive indicates that digital information resources play a positive moderating role, and vice versa for the negative moderating role.

#### 7.3.2. Digital Information Resource Measure

Digital information resources: we used the index of information accessibility to represent digital information resources. Information accessibility generally refers to the degree of information interaction or connectivity between individuals or regions [96]. According to theoretical analysis, the improvement of enterprise digitalization can help reduce enterprise information costs, improve enterprise specialization and cooperation levels, strengthen information sharing and knowledge spillover, and thus improve carbon emission intensity. Under the digital economy, information transmission features a digital encoding pathway. The information accessibility level of a region is closely related to its information infrastructure supply capacity and information resources. Based on the practice of existing studies, we constructed a comprehensive evaluation system of urban information accessibility from three dimensions: Internet information resources, digital industrialization information, and industrial digitalization information, as shown in Table 11. The entropy method is used for the calculation, and to avoid heteroscedasticity, we took the natural logarithm of the composite index (*lnaccess*). Finally, the city-enterprise matching is conducted according to the enterprise registration location.

#### 7.3.3. Moderating Effect Test

**Figure 2:**
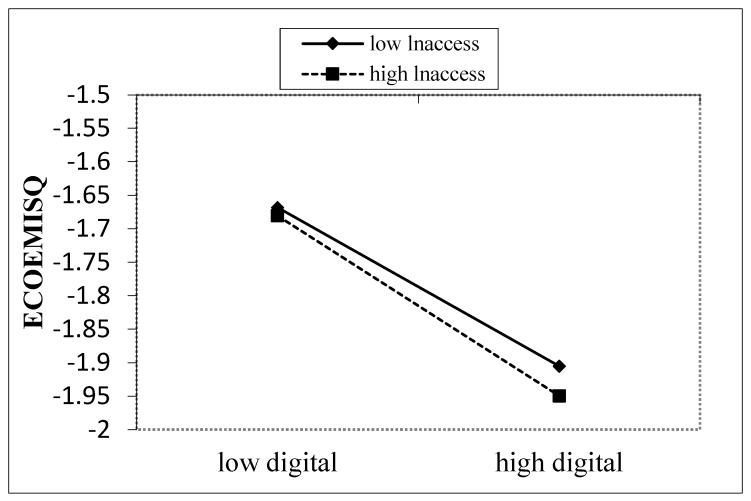
Reconciliation of digital information resources.

Table 12 reports the results of the estimation of the moderating effect. The coefficient of the interaction term in column (1) is negative and significant at the 1% statistical level, indicating that the inhibitory effect of digitalization on the carbon emission intensity of enterprises strengthens with the increase of digital information resources. Therefore, urban government department should provide digital information resources support for enterprises to accelerate digital transformation to reduce carbon emissions. Hypothesis H5 is verified, and digital information resources positively moderate the inhibitory effect of digital transition on carbon emission intensity. To show the moderating effect of digital information resources more visually, we created a map of the moderating effect (Figure 2). The moderating effect plot shows that the slope is steeper when the level of digital information resources increases. This indicates that the suppressive effect of enterprise digitalization on carbon emission intensity is strengthened.

Although existing studies analyzed the impact path of the micro-digitalization transition on carbon emissions from a micro perspective, they did not consider the regulatory role of macro-digital information resources [5,92]. This is because the digital information resources of the city where the enterprise is located will affect the efficiency of the enterprise’s digital application. For example, enterprises have carried out digital transformation, but the failure of broadband information facilities will reduce the efficiency of digital equipment operation. Therefore, it is necessary to explore the extent to which the influence path is affected by digital information resources. To further explore the path of moderating effects, we analyzed whether digital information resources have a moderating effect on the mediating variables. We replaced the explanatory variables in the moderating effect model with the mediating variables for testing. The estimated results are shown in columns (2) to (5) in Table 12. Because the coefficients of the interaction term in column (2) are consistent and significant, it can be seen that digital information resources can enhance the mitigating effect of digitalization of enterprises on enterprise information asymmetry. The coefficient of the interaction term in column (3) is negative but insignificant. The coefficient of the interaction term in column (4) shows that digital information resources can strengthen the promoting effect of digital transformation of enterprises on green technological innovation. The coefficient of the interaction term in column (5) shows that digital information resources strengthen the mitigating effect of digital transformation on corporate financing constraints. In summary, the moderating effect of digital information resources is mainly realized through three channels: financing constraint > green technology innovation > and information asymmetry. The moderating effect of digital information resources on energy use efficiency is not obvious, which is different from the existing research conclusions [97]. Therefore, the improvement of digital information resources can help strengthen the carbon reduction effect of digital transformation.

## 8. Conclusions and Implication

### 8.1. Conclusions

The text mining and IPCC method were used to measure the manufacturing enterprise digitalization and the level of enterprise carbon emission intensity from 2011 to 2021, respectively. This paper explores the influence and mechanism of digitalization on carbon intensity. We verify the reliability of the conclusion by using a series of methods such as the instrumental variable method and the GGM method. In addition, based on information theory, we comprehensively discuss the mechanism of digitalization—carbon reduction from four dimensions of information asymmetry, financing constraints, energy efficiency, and financing constraints—and explore the regulatory role of digital information resources in each influence path according to the characteristics of the digital economy. Finally, we further analyze the applicability conditions of digitalization-related carbon reduction. The conclusions are as follows: (1) digitalization can reduce enterprise carbon emission intensity significantly, and the influence shows the characteristic of a “marginal increase”. (2) The mechanism analysis shows that green technology innovation, financing constraints, energy efficiency, and information asymmetry play the role of intermediary mechanism. Interestingly, digital information resources positively moderate the positive effect of digitalization on carbon emission intensity through three paths: financing constraint, green technology innovation, information asymmetry; and (3) The influence has evident heterogeneity—as environmental regulation, financial development, executive education and R&D quality advance, the inhibitory effect of digitalization on enterprise carbon emission intensity increases.

### 8.2. Policy Implications

(1) Government departments increase the improvement of digital economy infrastructure. In particular, we will accelerate the integration of artificial intelligence, 5G, and the Internet of Things into the real economy. Enriching the city’s digital information resources and actively developing systems and methods to support the digital transformation of enterprises will help release digital technology dividends and realize low-carbon and high-quality development of manufacturing enterprises.

(2) Local governments actively respond to the national call to develop environmental regulation policies, vigorously develop the capital market, improve financial development, and provide richer channels for enterprise financing by raising R&D subsidy requirements to encourage enterprises to innovate with high quality. Always adhere to the strategy of developing the country through science and education, increase investment in education, expand postgraduate enrollment and provide intellectual support for the development of enterprises.

(3) The government should formulate incentive policies for enterprises’ digital transformation. The government reduces business operating costs through tax cuts and fee reductions, increases digital support for small and medium-sized enterprises and private enterprises, and ensures that enterprises have sufficient resources for digital transformation. Deepen the reform of the fiscal and taxation mechanism, gradually eliminate market segmentation, and provide institutional support for the flow of factors and information between regions and industries. Develop policies for the introduction of technical talents to improve the R&D capability of enterprises. Accelerate the digitalization reform of traditional finance and establish a multi-level capital market. The government should actively streamline financing procedures, and lower financing costs and financing thresholds to provide financial support for enterprises’ green R&D.

This paper is still inadequate: (1) Although this paper uses crawler technology to construct the digitalization index of enterprises by capturing the keywords of the digital economy in the annual reports of listed companies, which is scientific and reasonable. However, the annual report data are forward-looking and summarized, which may lead to bias in the estimation results.

The potential areas for further research: on the one hand, we need to consider whether the relationship between digitalization and carbon emissions is necessarily linear. Is there a nonlinear or threshold effect? This requires us to collect more complete data. On the other hand, whether the mechanism between digitalization and carbon emission has a superposition effect or extrusion effect is worthy of further analysis.

## Figures and Tables

**Table 1:** Coal conversion and carbon emission coefficient of various energy standards.

Types of Energy Consumption	A Standard Coal Conversion Factor	Carbon Emission Factor
coal	0.7143	0.7559
coke	0.9741	0.8550
raw	1.4286	0.5857
fuel	1.4286	0.6185
gas	1.4714	0.5538
kerosene	1.4714	0.5714
diesel	1.4571	0.6921
natural gas	13.3	0.4483
electric	0.1229	0.75

Data sources: China Energy Statistical Yearbook, 2006 IPCC National GHG Guidelines.

**Table 2:** Descriptive statistics of variables.

Variables	N	Mean	SD	Min	Max
ECOEMISQ	12,162	−0.0210	0.2182	−1.9150	2.8635
lndigital	12,162	0.4054	0.2473	0.0305	0.8673
lnage	12,162	2.8101	0.3549	1.0986	4.1271
lnsize	12,162	7.7936	1.1434	2.9957	12.3421
lnsale	12,162	21.4566	1.3547	15.4959	27.5118
lncash	12,162	0.4009	0.1974	0.0071	1.6356
HHI	12,162	0.1068	0.3088	0.0000	1.0000
TFP	12,162	0.9207	0.3258	0.0000	2.5578

**Table 3:** Baseline estimation tests.

Variables	(1)	(2)	(3)	(4)	(5)	(6)	(7)
lndigital	−0.2797 ***	−0.2683 ***	−1.0068 ***	−0.8266 **	−0.9274 ***	−0.9243 ***	−0.9382 ***
	(0.0583)	(0.0605)	(0.3636)	(0.3573)	(0.3512)	(0.3513)	(0.3512)
lnage		−0.0739	−0.0772	−0.1078	−0.0416	−0.0390	−0.0370
		(0.0768)	(0.0768)	(0.0772)	(0.0761)	(0.0760)	(0.0763)
lnsize			0.1045 **	0.0327	0.0549	0.0545	0.0561
			(0.0492)	(0.0474)	(0.0473)	(0.0473)	(0.0472)
lnsale				0.0713 ***	0.0824 ***	0.0826 ***	0.0822 ***
				(0.0119)	(0.0123)	(0.0122)	(0.0122)
lncash					−0.2906 ***	−0.2930 ***	−0.2915 ***
					(0.0501)	(0.0497)	(0.0494)
HHI						−0.1503 *	−0.1482 *
						(0.0812)	(0.0806)
TFP							−0.0205 **
							(0.0090)
Constant	0.6635 ***	0.8250 ***	1.5292 ***	0.2845	0.0280	0.0322	0.0504
	(0.1180)	(0.1912)	(0.4084)	(0.4207)	(0.4178)	(0.4174)	(0.4166)
i.company	√	√	√	√	√	√	√
i.year	√	√	√	√	√	√	√
Obs	12,162	12,162	12,162	12,162	12,162	12,162	12,162
R-squared	0.0227	0.0230	0.0235	0.0293	0.0377	0.0380	0.0385

Note: robust standard errors in parentheses, *** *p* < 0.01, ** *p* < 0.05, * *p* < 0.1. The same is below.

**Table 4:** Robustness test.

Variables	(1)	(2)	(3)	(4)	(5)
	Outlier Value Handling	Alternative Estimation Method	Change of Independent Variable	Replacing the Explained Variable	Missing Variable Handling
lndigital	−1.5197 ***	−0.6603 ***	−2.6411 ***	−0.0011 **	−0.4319 *
	(0.3718)	(0.2304)	(0.6752)	(0.0017)	(0.2319)
lnage	−0.1116 ***	−0.0060	−0.3389 ***	−0.0239	0.0127
	(0.0311)	(0.0102)	(0.0845)	(0.0807)	(0.0102)
lnsize	0.1245 **	0.0061	0.2882 ***	−0.0753 ***	−0.0437
	(0.0491)	(0.0299)	(0.0897)	(0.0120)	(0.0297)
lnsale	0.0823 ***	0.0791 ***	0.0200	0.0847 ***	0.1019 ***
	(0.0076)	(0.0058)	(0.0168)	(0.0126)	(0.0050)
lncash	−0.2389 ***	−0.2635 ***	−2.7523 ***	−0.2878 ***	−0.3977 ***
	(0.0273)	(0.0241)	(0.0745)	(0.0502)	(0.0532)
HHI	−0.1172 *	−0.0087	−0.3099 *	−0.1497 *	0.0013
	(0.0651)	(0.0124)	(0.1802)	(0.0815)	(0.0117)
TFP	−0.0118 ***	−0.0063	−0.0168 *	−0.0215 **	−0.0144 **
	(0.0036)	(0.0074)	(0.0087)	(0.0091)	(0.0072)
rd					0.0017
					(0.0056)
lev					0.0653 **
					(0.0255)
Constant	0.8418 **	−0.1812	5.4679 ***	−0.9308 ***	−0.7329 ***
	(0.4256)	(0.2750)	(0.8179)	(0.2663)	(0.2726)
i.company	√	√	√	√	√
i.year	√	√	√	√	√
Obs	12,162	12,162	12,162	13,182	12,162
R-squared	0.1917		0.5514	0.0363	0.0999

*** *p* < 0.01, ** *p* < 0.05, * *p* < 0.1.

**Table 5:** Endogeneity test.

Variables	(1)	(2)	(3)	(4)
	First Stage	2SLS	SYSGMM	DiffGMM
lndigital		−10.9066 **	−10.0944 ***	−16.3673 *
		(5.2770)	(3.3194)	(9.3582)
lnage	0.0002	−0.0327	−0.0647	−2.1088
	(0.0013)	(0.0474)	(0.0737)	(1.7432)
lnsize	0.1387	1.4398 **	1.2542 ***	2.2141 *
	(0.0003)	(0.7325)	(0.4396)	(1.3198)
lnsale	−0.0019	0.0637 ***	0.0404 **	0.0566
	(0.0003)	(0.0134)	(0.0201)	(0.0398)
lncash	−0.0031	−0.3214 ***	−0.2411 ***	−0.1873 ***
	(0.0008)	(0.0345)	(0.0447)	(0.0618)
HHI	0.0009	−0.1335	−0.0079	0.0124
	(0.0024)	(0.0853)	(0.0363)	(0.0430)
TFP	−0.0005	−0.0263 ***	−0.0211	−0.0180 *
	(0.0002)	(0.0101)	(0.0144)	(0.0102)
instrument	−0.0035 ***			
	(0.0005)			
F-value in Phase I		19.01		
AR2			*p* = 0.242	*p* = 0.256
Hansen			*p* = 0.761	*p* = 0.795
Constant	1.0697 ***	10.0381 *	10.1133 ***	
	(0.0117)	(5.2906)	(3.6504)	
i.company	√	√	√	√
i.year	√	√	√	√
Obs	12,162	10,788	12,162	10,853
R-squared	0.9854	0.0703	0.5049	-

*** *p* < 0.01, ** *p* < 0.05, * *p* < 0.1.

**Table 6:** Environmental regulation and financial development.

Variables	(1)	(2)	(3)	(4)
	Regulated Cities		Level of Financial Development	
	Yes	No	Low	High
lndigital	−2.1298 ***	−0.6405 *	−1.4146	−1.8248 ***
	(0.4199)	(0.6290)	(0.9357)	(0.6009)
lnage	0.0087	−0.1468 *	0.3128	0.0154
	(0.0983)	(0.0853)	(0.3499)	(0.1049)
lnsize	0.2075 ***	−0.1542 *	−0.2321 *	0.1311
	(0.0569)	(0.0847)	(0.1314)	(0.0834)
lnsale	0.0822 ***	0.0927 ***	0.0340	0.1042 ***
	(0.0176)	(0.0191)	(0.0410)	(0.0203)
lncash	−0.2782 ***	−0.2932 ***	−0.4032 ***	−0.2495 ***
	(0.0709)	(0.0628)	(0.1480)	(0.0762)
HHI	−0.0734 **	−0.1624 *		
	(0.0370)	(0.0855)	-	-
TFP	−0.0276 **	−0.0075	−0.0543	−0.0075
	(0.0118)	(0.0153)	(0.0370)	(0.0086)
Constant	1.2028 **	−1.5078 **	−2.2932 *	0.5616
	(0.5138)	(0.6887)	(1.2198)	(0.7910)
i.company	√	√	√	√
i.year	√	√	√	√
Obs	8973	3189	6638	5524
R-squared	0.0388	0.0416	0.0189	0.1264

*** *p* < 0.01, ** *p* < 0.05, * *p* < 0.1.

**Table 7:** Quantile test of carbon emission intensity.

Variables	(1)	(2)	(3)	(4)
	25%	50%	75%	99%
lndigital	−0.4015	−0.8706 **	−1.4045 **	−2.4876 **
	(0.3788)	(0.3462)	(0.5730)	(1.2363)
lnage	−0.0949	−0.0964 *	−0.0981	−0.1016
	(0.0592)	(0.0540)	(0.0895)	(0.1929)
lnsize	−0.0068	0.0406	0.0946	0.2041
	(0.0495)	(0.0452)	(0.0749)	(0.1615)
lnsale	0.0880 ***	0.0918 ***	0.0962 ***	0.1050 ***
	(0.0096)	(0.0087)	(0.0145)	(0.0313)
lncash	−0.3173 ***	−0.2849 ***	−0.2481 ***	−0.1733
	(0.0446)	(0.0408)	(0.0675)	(0.1456)
HHI	−0.1332 **	−0.1383 ***	−0.1442 *	−0.1561
	(0.0575)	(0.0525)	(0.0870)	(0.1875)
TFP	−0.0099	−0.0099	−0.0099	−0.0099
	(0.0111)	(0.0101)	(0.0168)	(0.0362)
i.company	√	√	√	√
i.year	√	√	√	√
Obs	12,162	12,162	12,162	12,162

*** *p* < 0.01, ** *p* < 0.05, * *p* < 0.1.

**Table 8:** Executive education background and R&D quality.

Variables	(1)	(2)	(3)	(4)
	Educational Background		R&D Quality	
	Master’s Degree or Above	Bachelor’s Degree or Below	Low Quality	High Quality
lndigital	−3.5826 **	−1.6485	2.0943 *	−1.2839 ***
	(1.5505)	(1.3654)	(0.6498)	(0.3714)
lnage	0.0471	−0.1504	0.1559	−0.0260
	(0.1034)	(0.1694)	(0.4665)	(0.0754)
lnsize	0.3347 *	0.2068	−0.3623 ***	0.1090 **
	(0.1760)	(0.2357)	(0.0918)	(0.0500)
lnsale	0.1008 ***	0.0627 **	0.0851 **	0.0851 ***
	(0.0142)	(0.0247)	(0.0376)	(0.0126)
lncash	−0.1987 ***	−0.4016 ***	0.2360	−0.3687 ***
	(0.0656)	(0.1133)	(0.1606)	(0.0485)
HHI	−0.1224 **	−0.3047		
	(0.0618)	(0.2664)		
TFP	−0.0201 **	−0.0058	−0.0096	−0.0192 *
	(0.0098)	(0.0187)	(0.0167)	(0.0100)
Constant	2.6375	1.1563	−3.7362 ***	0.2666
	(1.7963)	(1.1115)	(1.1255)	(0.4380)
i.company	√	√	√	√
i.year	√	√	√	√
Obs	9234	2928	1423	10,739
R-squared	0.0280	0.0737	0.0255	0.0486

*** *p* < 0.01, ** *p* < 0.05, * *p* < 0.1.

**Table 9:** Information asymmetry and energy efficiency.

Variables	(1)	(2)	(3)	(4)	(5)	(6)
	lnasymme	ECOEMISQ	ECOEMISQ	lnenergy	ECOEMISQ	ECOEMISQ
lndigital	−0.1589 *		−0.9012 ***	−0.4204 ***		−0.8609 **
	(0.0872)		(0.3116)	(0.0253)		(0.3563)
lnasymme		0.2345 ***	0.2328 ***			
		(0.0338)	(0.0338)			
lnenergy					0.2334 ***	0.1839 *
					(0.0855)	(0.0947)
lnage	−0.0439 ***	−0.0270	−0.0267	0.0235 ***	−0.0428	−0.0413
	(0.0127)	(0.0453)	(0.0453)	(0.0037)	(0.0762)	(0.0760)
lnsize	0.0133	−0.0721 ***	0.0530	0.0530 ***	−0.0729 ***	0.0463
	(0.0124)	(0.0094)	(0.0442)	(0.0036)	(0.0116)	(0.0479)
lnsale	0.0046 *	0.0828 ***	0.0811 ***	−0.0095 ***	0.0860 ***	0.0840 ***
	(0.0024)	(0.0087)	(0.0087)	(0.0007)	(0.0125)	(0.0124)
lncash	0.0502 ***	−0.3006 ***	−0.3032 ***	−0.0029	−0.2883 ***	−0.2910 ***
	(0.0082)	(0.0294)	(0.0294)	(0.0024)	(0.0494)	(0.0494)
HHI	−0.0829 ***	−0.1300	−0.1289	−0.0060	−0.1480 *	−0.1471 *
	(0.0227)	(0.0812)	(0.0812)	(0.0066)	(0.0810)	(0.0804)
TFP	0.0008	−0.0202 **	−0.0207 **	−0.0007	−0.0199 **	−0.0204 **
	(0.0026)	(0.0091)	(0.0091)	(0.0007)	(0.0090)	(0.0090)
Constant	0.3933 ***	−0.9444 ***	−0.0411	0.6183 ***	−0.9356 ***	−0.0633
	(0.1007)	(0.1791)	(0.3600)	(0.0292)	(0.2645)	(0.4367)
i.company	√	√	√	√	√	√
i.year	√	√	√	√	√	√
Obs	12,162	12,162	12,162	12,162	12,162	12,162
R-squared	0.0113	0.0418	0.0425	0.0731	0.0380	0.0387

*** *p* < 0.01, ** *p* < 0.05, * *p* < 0.1.

**Table 10:** Green technology innovation.

Variables	(1)	(2)	(3)	(4)	(5)	(6)
	lngpatent	ECOEMISQ	ECOEMISQ	lnSA	ECOEMISQ	ECOEMISQ
lndigital	0.7386 ***		−0.0722 *	−0.5373 **		−0.3466 *
	(0.2696)		(0.2998)	(0.2243)		(0.2941)
lngpatent		−1.3677 ***	−1.3681 ***			
		(0.1151)	(0.1157)			
lnSA					1.1031 ***	1.1009 ***
					(0.0342)	(0.0346)
lnage	0.1361 ***	0.1492 **	0.1493 **	−0.1403 ***	0.1176 *	0.1175 *
	(0.0311)	(0.0737)	(0.0737)	(0.0231)	(0.0697)	(0.0696)
lnsize	−0.0306	0.0242 *	0.0142	0.0252	−0.0197 **	0.0283
	(0.0364)	(0.0128)	(0.0366)	(0.0300)	(0.0094)	(0.0389)
lnsale	−0.0742 ***	−0.0194	−0.0193	0.0387 ***	0.0402 ***	0.0396 ***
	(0.0078)	(0.0137)	(0.0135)	(0.0058)	(0.0102)	(0.0099)
lncash	0.2136 ***	0.0004	0.0007	−0.1451 ***	−0.1304 ***	−0.1318 ***
	(0.0289)	(0.0355)	(0.0359)	(0.0205)	(0.0388)	(0.0390)
HHI	0.1334 *	0.0344	0.0344	−0.0831	−0.0570	−0.0567
	(0.0747)	(0.0280)	(0.0279)	(0.0747)	(0.0564)	(0.0565)
TFP	0.0113 ***	−0.0051	−0.0050	−0.0086 ***	−0.0108	−0.0110
	(0.0036)	(0.0073)	(0.0073)	(0.0029)	(0.0086)	(0.0086)
Constant	−0.2001	−0.1512	−0.2233	1.1198 ***	−1.5309 ***	−1.1824 ***
	(0.3224)	(0.1801)	(0.2943)	(0.2619)	(0.2162)	(0.3212)
i.company	√	√	√	√	√	√
i.year	√	√	√	√	√	√
Obs	12,162	12,162	12,162	12,162	12,162	12,162
R-squared	0.1827	0.2699	0.2699	0.1476	0.1278	0.1279

*** *p* < 0.01, ** *p* < 0.05, * *p* < 0.1.

**Table 11:** Comprehensive index system of information accessibility.

Level 1 Indicators	Level 2 Indicators	
Internet information resources	Number of domain names with ten thousand people (person/ten thousand)	+
	Number of mobile Internet users (10,000)	+
	Internet access ports (10,000)	+
	Internet penetration rate	+
	Mobile phone penetration rate	+
Digital industrialization information	Sales revenue of software technology (ten thousand)	+
	E-commerce sales (10,000)	+
	E-commerce purchase amount (10,000)	+
	Number of e-commerce enterprises (10,000)	+
Industrial digitalization information	Digital-inclusive Financial Development Index	+
	Number of 5G patents authorized	+
	Number of industrial Internet patents authorized	+
	Number of e-commerce patents authorized	+

Note: the plus sign "+" in the table represents a positive impact on the composite index.

**Table 12:** Moderating effect of digital information resources.

Variables	(1)	(2)	(3)	(4)	(5)
	ECOEMISQ	lnasymme	lnenergy	lngpatent	lnSA
lndigital	−0.6624 *	−0.1235	−0.4479	0.6038 **	−0.3225
	(0.3957)	(0.1960)	(0.3677)	(0.2897)	(0.2346)
lndigital_lnaccess	−0.2941 **	−0.0405 *	−0.0259	0.1479 **	−0.2246 ***
	(0.1446)	(0.0627)	(0.0321)	(0.0906)	(0.0721)
lnaccess	0.5245 *	0.0605	−0.0611	−0.2462	0.4198 ***
	(0.3093)	(0.1315)	(0.0713)	(0.1912)	(0.1518)
lnage	−0.0209	−0.0411 *	0.0229 **	0.1272 ***	−0.1290 ***
	(0.0785)	(0.0232)	(0.0097)	(0.0314)	(0.0234)
lnsize	0.0468	0.0125	0.0543	−0.0265	0.0174
	(0.0490)	(0.0255)	(0.0464)	(0.0368)	(0.0300)
lnsale	0.0813 ***	0.0044	−0.0094	−0.0738 ***	0.0380 ***
	(0.0123)	(0.0050)	(0.0059)	(0.0078)	(0.0057)
lncash	−0.2842 ***	0.0514 ***	−0.0033	0.2095 ***	−0.1399 ***
	(0.0489)	(0.0159)	(0.0035)	(0.0288)	(0.0205)
HHI	−0.1475 *	−0.0828 **	−0.0060	0.1331 *	−0.0826
	(0.0804)	(0.0386)	(0.0066)	(0.0748)	(0.0737)
TFP	−0.0211 **	0.0007	−0.0006	0.0116 ***	−0.0092 ***
	(0.0093)	(0.0028)	(0.0004)	(0.0036)	(0.0029)
Constant	−0.3972	0.3425	0.6716	0.0086	0.7601 ***
	(0.5229)	(0.2388)	(0.4919)	(0.3725)	(0.2938)
i.company	√	√	√	√	√
i.year	√	√	√	√	√
Obs	12,162	12,162	12,162	12,162	12,162
R-squared	0.0394	0.0119	0.0742	0.1851	0.1524

*** *p* < 0.01, ** *p* < 0.05, * *p* < 0.1.

## Data Availability

The data used in this paper are all from the CSMAR database, specific website: https://www.gtarsc.com/. However, this article does not produce any datasets.
